# Oxidative stress induces monocyte‐to‐myofibroblast transdifferentiation through p38 in pancreatic ductal adenocarcinoma

**DOI:** 10.1002/ctm2.41

**Published:** 2020-06-04

**Authors:** Xin Huang, Chaobin He, Xin Hua, Anna Kan, Yize Mao, Shuxin Sun, Fangting Duan, Jun Wang, Peng Huang, Shengping Li

**Affiliations:** ^1^ State Key Laboratory of Oncology in South China, Collaborative Innovation Center for Cancer Medicine Sun Yat‐sen University Cancer Center Guangzhou People's Republic of China; ^2^ Department of Experimental Research Sun Yat‐sen University Cancer Center Guangzhou People's Republic of China; ^3^ Department of Pancreatobiliary Surgery Sun Yat‐sen University Cancer Center Guangzhou People's Republic of China; ^4^ Department of Medical Oncology Sun Yat‐sen University Cancer Center Guangzhou People's Republic of China; ^5^ Department of Hepatic Surgery Sun Yat‐sen University Cancer Center Guangzhou People's Republic of China

**Keywords:** monocyte‐to‐myofibroblast transdifferentiation, oxidative stress, p38, p53, pancreatic ductal adenocarcinoma

## Abstract

**Background:**

Cancer‐associated fibroblasts (CAFs) are among the most prominent cells during the desmoplastic reaction in pancreatic ductal adenocarcinoma (PDAC). However, CAFs are heterogeneous and the precise origins are not fully elucidated. This study aimed to explore whether monocytes can transdifferentiate into fibroblasts in PDAC and evaluate the clinical significance of this event.

**Methods:**

CD14^+^ monocytes were freshly isolated from human peripheral blood. Immunofluorescence, reverse transcription‐quantitative PCR, western blot, flow cytometry and enzyme‐linked immunosorbent assay were used to detect the expression of αSMA, fibronectin, and other relevant molecules. In addition, latex beads with a mean particle size of 2.0 µm were used to assess the phagocytic capacity. Moreover, RNA sequencing (RNA‐seq) was performed to identify the differences induced by H_2_O_2_ and the underlying mechanisms.

**Results:**

Immunofluorescence identified αSMA and fibroblast‐specific protein 1 expression by tumor‐associated macrophages in PDAC. The in vitro experiment revealed that oxidative stress (H_2_O_2_ or radiation) induced monocyte‐to‐myofibroblast transdifferentiation (MMT), as identified by upregulated αSMA expression at both the RNA and protein levels. In addition, compared with freshly isolated monocytes, human monocyte‐derived macrophages increased fibronectin expression. RNA‐seq analysis identified p53 activation and other signatures accompanying this transdifferentiation; however, the p53 stabilizer nutlin‐3 induced αSMA expression through reactive oxygen species generation but not through the p53 transcription/mitochondria‐dependent pathway, whereas the p38 inhibitor SB203580 could partially inhibit αSMA expression. Finally, MMT produced a unique subset of CAFs with reduced phagocytic capacity that could promote the proliferation of pancreatic cancer cells.

**Conclusions:**

Oxidative stress in the tumor microenvironment could induce MMT in PDAC, thus inducing reactive stroma, modulating immunosuppression, and promoting tumor progression. Reducing oxidative stress may be a promising future therapeutic regimen.

## Graphical abstract

Monocytes in human blood are recruited into PDAC tissues. Low intracellular levels of H_2_O_2_ are important for fibronectin mRNA synthesis, protein expression and secretion. Oxidative stress can reduce the phagocytic capacity and simultaneously activate the p38‐MAPK signaling pathway, thus inducing αSMA mRNA and protein expression (monocyte‐to‐myofibroblast transdifferentiation). However, these cells do not express Col1. In addition, supernatant from these cells can stimulate pancreatic cancer cell (AsPC‐1) proliferation.

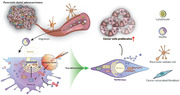

**Abbreviations**
CAFs: cancer‐associated fibroblasts
CCK‐8: cell counting kit‐8
Col1: collagen1
DEGs: differentially expressed genes
DMSO: dimethyl sulfoxide
ECM: extracellular matrix
ELISA: enzyme‐linked immunosorbent assay
FAP: fibroblast activation protein
FPKM: fragments per kilobase of exon per million fragments mapped
FSP1: fibroblast‐specific protein 1
HMDMs: human monocyte‐derived macrophages
iCAFs: inflammatory cancer‐associated fibroblasts
IF: immunofluorescence
KEGG: Kyoto Encyclopedia of Genes and Genomes
MMT: monocyte‐to‐myofibroblast transdifferentiation
MSCs: mesenchymal stem cells
NFs: normal fibroblasts
PBMCs: peripheral blood mononuclear cells
PDAC: pancreatic ductal adenocarcinoma
PSCs: pancreatic stellate cells
ROS: reactive oxygen species
RT‐qPCR: reverse transcription‐quantitative polymerase chain reaction
TAMs: tumor‐associated macrophages
WB: western blotMAPK,mitogen-activated protein kinase

## 1 INTRODUCTION

Pancreatic ductal adenocarcinoma (PDAC) is a notoriously aggressive cancer with a 5‐year survival rate of less than 10% and is projected to be the second leading cause of cancer deaths by 2030.^1^, ^2^ Due to local invasion and distant metastasis, only 20% of patients are candidates for surgical resection, which remains the sole potentially curative option because of relative resistance to chemotherapy, radiotherapy, or immunotherapy.^3^, ^4^

PDAC is characterized by an extensive fibrotic response. The main components of the stroma are cancer‐associated fibroblasts (CAFs), tumor‐associated macrophages (TAMs), vascular endothelial cells, extracellular matrix (ECM), and so forth. CAFs are characterized by the expression of αSMA, fibroblast‐specific protein 1 (FSP1), fibroblast activation protein (FAP), collagen1 (Col1), or fibronectin, and αSMA is the most classical and acceptable marker, which is also expressed by vascular smooth muscle cells and associated with cell contractility.^5^ CAFs in PDAC are thought to promote cancer initiation and progression, and to induce chemoresistance by secreting chemokines and ECM, which impedes the delivery of therapeutic agents.^6^ However, targeting CAFs leads to PDAC disease progression in both humans and mice.^7^, ^8^ In addition, other researchers have reported that stromal elements act to suppress rather than support pancreatic cancer development.^9^ These studies highlighted the heterogeneity of CAFs, which might be ascribed to the numerous potential origins of CAFs.^10^ In other cancers, local proliferation of resident fibroblasts, epithelial‐to‐mesenchymal transition, and endothelial‐to‐mesenchymal transition have been reported to contribute to the CAFs population.^11^ In PDAC, quiescent pancreatic stellate cells (PSCs) and mesenchymal stem cells (MSCs) have been reported to contribute to the CAFs pool.^12^, ^13^

TAMs have an important role in promoting PDAC progression.^14^ Human blood monocytes are recruited into PDAC tissues, where they differentiate into macrophages. These human monocyte‐derived macrophages (HMDMs) face an unfavorable environment characterized by accumulation of reactive oxygen species (ROS), which are generated as byproducts of intracellular oxygen metabolism or in response to exogenous stimuli, including chemotherapeutics and ionizing radiation. Generally, moderate levels of ROS act as signals to promote cell survival and function, whereas a severe increase in ROS levels can induce cell death.^15^ Studies have shown that ROS are essential for monocyte survival and differentiation.^16^ Other researchers used 200 µM H_2_O_2_ to induce oxidative stress and found that ROS induced CCR5 expression by monocytes.^17^ Another study demonstrated that 100 µM H_2_O_2_ stimulated monocytes/macrophages to actively release HMGB1.^18^ Recently, other reports have indicated that ROS induce PD‐L1 expression by TAMs in breast cancer.^19^

Transdifferentiation is a process of lineage reprogramming whereby one terminally differentiated somatic cell directly transforms into another mature somatic cell.^20^, ^21^ Tanabe et al recently reported that human adult peripheral blood T lymphocytes can be directly converted into fully functional neurons.^22^ Other reports have indicated that pre‐B cells,^23^ smooth muscle cells,^24^ and fibroblasts^25^ can transdifferentiate into macrophages.

The density of macrophages correlates with that of αSMA^+^ fibroblasts and with Col1 deposition in PDAC.^26^ Numerous studies have shown that macrophages are located very near myofibroblasts and could regulate fibrosis by producing profibrotic mediators, including TGF‐β1.^27^, ^28^ In addition, other studies have reported that subsets of TAMs express FSP1, FAP, or Col1, which are relatively specific to fibroblasts.^29^, ^30^, ^31^ Research on fibrotic diseases, such as renal fibrosis, has indicated that bone marrow‐derived monocytes can transdifferentiate into myofibroblasts via a process termed monocyte‐to‐myofibroblast transdifferentiation (MMT).^32^ However, MMT has not been explored clearly in cancers, and its molecular mechanism has not been fully elucidated.

ROS have been reported to promote myofibroblast differentiation and tumor spreading in cancers.^33^ In this study, we demonstrated for the first time that cellular oxidative stress can induce human monocytes to transdifferentiate into CAFs in PDAC, thus promoting fibrosis, hampering the immune reaction, and promoting tumor progression.

## 2 METHODS

### 2.1 Isolation and culture of cells

CD14^+^ monocytes were enriched from peripheral blood mononuclear cells (PBMCs) as previously described.^34^ Monocytes were cultured in serum‐free DMEM for 30 min in 5% CO_2_ at 37°C in order to allow adhesion, and then cultured in DMEM containing 10% human AB serum (100‐318; Gemini). Medium was collected or replaced every 3‐4 days.

Human PDAC cell lines (AsPC‐1, BxPC‐3, CFPAC‐1, and PANC‐1) and HPDE6‐C7 were cultured, and the cell supernatant was collected for 24 h.^34^

PSCs were isolated as others,^35^ including CAFs from tumor tissue and normal fibroblasts (NFs) from matching normal tissue of the same patient. Cell populations between passage 3 and 6 were used.

**Highlights**

1. Myofibroblast‐specific markers (αSMA and FSP1) were expressed by TAMs in pancreatic ductal adenocarcinoma.
2. Oxidative stress induced MMT in a time‐ and concentration‐dependent manner by activating p38‐MAPK pathway.
3. MMT defined a unique subset of CAFs with reduced phagocytic capacity.

### 2.2 Drugs treatment

Nutlin‐3 was purchased from Selleck Chemicals (S1061; USA), dissolved in dimethyl sulfoxide (DMSO), and stored at –20°C. TGF‐β1 (100‐21; Peprotech, USA) or H_2_O_2_ (323381; Sigma Aldrich) was added into the culture medium. Considering the rapid metabolism of H_2_O_2_, exogenous H_2_O_2_ was added twice a day. To inhibit p53 transcription or translocation activity, monocytes were pretreated with PFT‐α (S5791; Selleck) or PFT‐μ (S2930; Selleck), respectively. The p38 specific inhibitor, SB203580 (S1076; Selleck), was added 30 min prior to the addition of other drugs. In addition, 100 U/mL catalase (C1345; Sigma Aldrich) was added daily to eradicate the intracellular H_2_O_2_.

### 2.3 Radiation

Cells were irradiated (0, 2, 4, 8, or 16 Gy) as previously described.^36^

### 2.4 Cell viability

Cell viability was examined by a Cell Counting Kit‐8 (CCK‐8; Dojindo, Japan) assay to investigate an appropriate concentration of H_2_O_2_ or radiation dose for monocytes cultured in vitro. A total of 1.25 × 10^5^ cells were plated into 96‐well plates giving a final volume of 200 µL of culture medium with 0, 50, 100, 200, 300, 500, or 1000 µM H_2_O_2_; or cells were irradiated with 0, 2, 4, or 8 Gy. After incubation for the indicated time (1, 3, or 7 days), the cultured medium was removed; we added CCK‐8 reagent (incubation at 37°C for 2 h) and measured the absorption at 450 nm (Bio‑Rad Laboratories, USA).

The cell viability of cancer cells exposed to different treatments was detected as described above, with 2000‐3000 cells plated into 96‐well plates and cultured for the indicated time. For the supernatant, 2 × 10^5^ monocytes were treated with 100 µM H_2_O_2_ (twice a day) or not for 7 days in 6‐well plates, and then the medium was changed to complete medium supplemented with 10% fetal bovine serum. Similar to other study,^37^ after 36 h, supernatant was centrifuged and collected.

### 2.5 Flow cytometry

Freshly isolated monocytes were stained with PE/Cy7‐conjugated anti‐CD14 (301814; Biolegend).^34^ For ROS measurement, monocytes were seeded at 0.5 × 10^6^ cells/mL and treated with H_2_O_2_, irradiation, nutlin‐3, or DMSO. After the indicated time periods, cells were washed with PBS and resuspended with 10 µM H2DCFDA (D399; Invitrogen) in the dark for 30 min at 37°C. Cells were then washed for twice. Fluorescence was measured and data of mean fluorescence intensity were analyzed by Kaluza (Beckman Coulter, USA).

### 2.6 Reverse transcription‐quantitative polymerase chain reaction

Reverse transcription‐quantitative polymerase chain reaction (RT‐qPCR) was performed as described previously.^34^ The primer sequences were listed in the Table S1.

### 2.7 Western blot

We performed western blot (WB) analysis as previously described^36^ using an ECL kit (4AW011; purchased from 4A Biotech Co., Ltd) and the following antibodies: anti‐GAPDH (1:5000, 60004‐1‐Ig; Proteintech, USA), anti‐αSMA (1:1000, ab124964; Abcam, UK), anti‐fibronectin (1:1000, ab45688; Abcam, UK), anti‐MDM2 (1:1000, #86934; Cell Signaling Technology, USA), anti‐t‐p53 (1:1000, 10442‐1‐AP; Proteintech, USA), anti‐Col1 (1:1000, #84336; Cell Signaling Technology, USA), anti‐vimentin (1:1000, #5741; Cell Signaling Technology, USA), anti‐CD68 (1:5000, 25747‐1‐AP; Proteintech, USA), anti‐p‐p38 (1:1000, #4511; Cell Signaling Technology, USA), anti‐t‐p38 (1:1000, #8690; Cell Signaling Technology, USA), anti‐p21 (1:1000, ab109520; Abcam, UK), and nti‐FSP1 (1:1000, ab124805; Abcam, UK).

### 2.8 Cell and tissue immunofluorescence

Cell or tissue immunofluorescence (IF) staining was performed as previously reported^34^, ^36^; cells were incubated with anti‐αSMA (1:300), FSP1 (1:100), or anti‐CD68 (1:100, ZM‐0464; Zhongshan Golden Bridge Biotechnology Co., Beijing, China) overnight at 4°C.

### 2.9 Enzyme‐linked immunosorbent assay

Human fibronectin from cell culture supernatant was detected by enzyme‐linked immunosorbent assay (ELISA) (CSB‐E04551h; Cusabio Biotech, Houston, TX, USA); 96‐well plates were read at 450 and 540 nm and data were analyzed as others.^38^

### 2.10 Phagocytosis of latex beads

Human monocytes were incubated with or without 100 µM H_2_O_2_ for the indicated time. Cells were starved for 12 h and incubated with latex beads (L3030; Sigma) with a mean particle size of 2.0 µm for 24 h; they were processed as aforementioned cell IF. When cells phagocyted numerous latex beads, they became red in fluorescent microscope. Phagocytotic activity was calculated as the number of cells phagocyting beads divided by the number of nuclei.^24^

### 2.11 RNA sequencing library preparation and transcriptomic analysis

Monocytes were cultured with or without 100 µM H_2_O_2_ for 7 days. Respective total RNAs were extracted and random primers were used to generate cDNA. Sequencing was carried out using an Illumina HiSeq 4000 Sequencing System for 150 cycles (KangChen Bio‐tech Inc, Shanghai, China). After the data preprocessing, fragments per kilobase of exon per million fragments mapped (FPKM) and differentially expressed genes (DEGs) were then calculated,^39^, ^40^ with the FPKM ≥ 0.5 (Cuffquant) considered statistically significant. Furthermore, Kyoto Encyclopedia of Genes and Genomes (KEGG) tool was used for pathway analysis of the DEGs.

### 2.12 Statistical analysis

Data are expressed as the means ± standard errors of the mean. We used independent or paired *t*‐test or a Wilcoxon matched‐pairs test for comparison between groups, and one‐way or two‐way ANOVA with the Bonferroni's post hoc test for multiple comparisons. GraphPad prism 7 was used for statistical analysis using two tailed tests and *P* < .05 was considered statistically significant.

## 3 RESULTS

### 3.1 Expression of myofibroblast markers by TAMs in PDAC

PDAC is characterized by extensive fibrosis, and studies have widely adopted αSMA assessment approaches to evaluate the degree of fibrosis in cancers, including PDAC.

**FIGURE 1:**
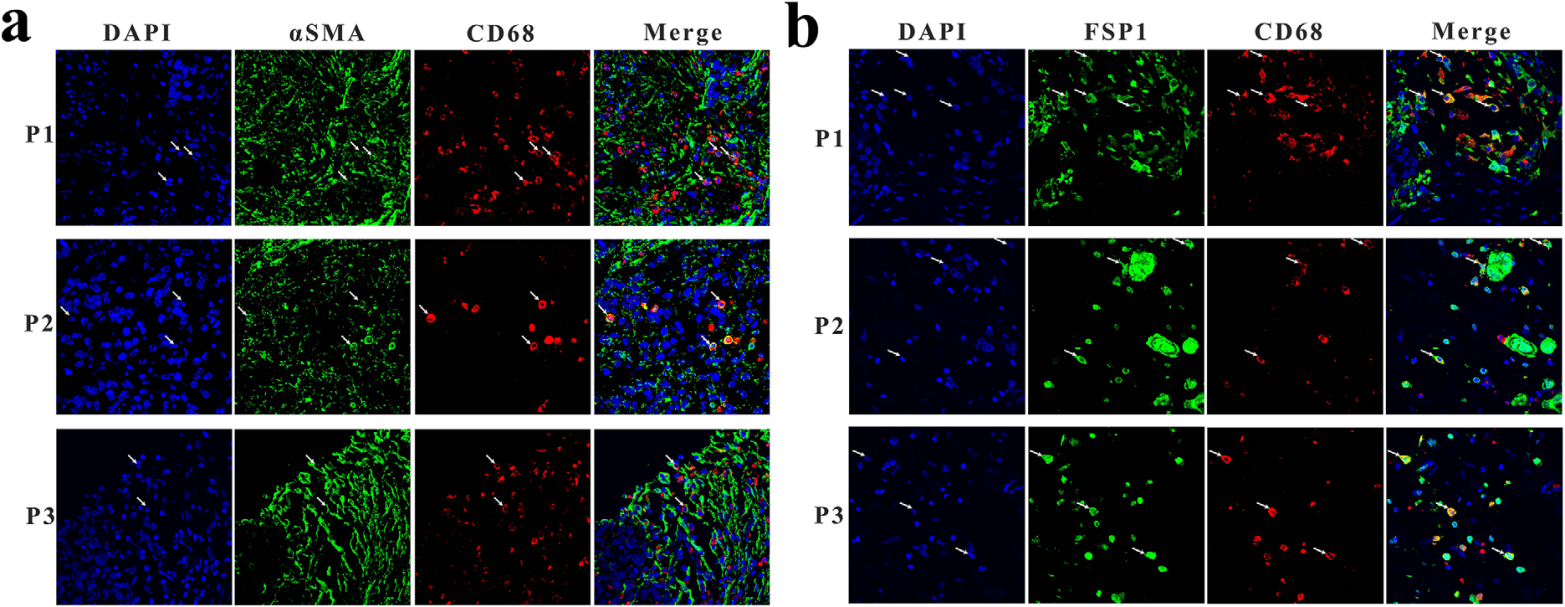
Expression of myofibroblast markers by TAMs in PDAC. A, Immunofluorescence staining for αSMA (green), CD68 (red), and DAPI (blue, for nuclear staining) in PDAC tissues of three patients (P1, P2, and P3). B, Immunofluorescence staining for FSP1 (green), CD68 (red), and DAPI (blue, for nuclear staining) in PDAC tissues of three patients (P1, P2, and P3) Abbreviations: TAMs, tumor associated macrophages; PDAC, pancreatic ductal adenocarcinoma; DAPI, 4′,6‐diamidino‐2‐phenylindole; FSP1, fibroblast‐specific protein 1.

To investigate whether monocytes/macrophages can transdifferentiate into (myo)fibroblasts in PDAC, we first examined the distribution of TAMs and CAFs in paraffin‐embedded tissues derived from PDAC patients. We adopted two‐color IF and detected intermediate cells that uniquely co‐expressed a pan‐macrophage marker (CD68) and a myofibroblast marker (αSMA) in PDAC tissue (Figure 1A) but not in normal pancreas tissue (data not shown). In addition, we detected colocalization of CD68 and FSP1 in PDAC tissue, suggesting that monocytes/macrophages contribute to FSP1 expression (Figure 1B).

### 3.2 Oxidative stress induced MMT in vitro

**FIGURE 2:**
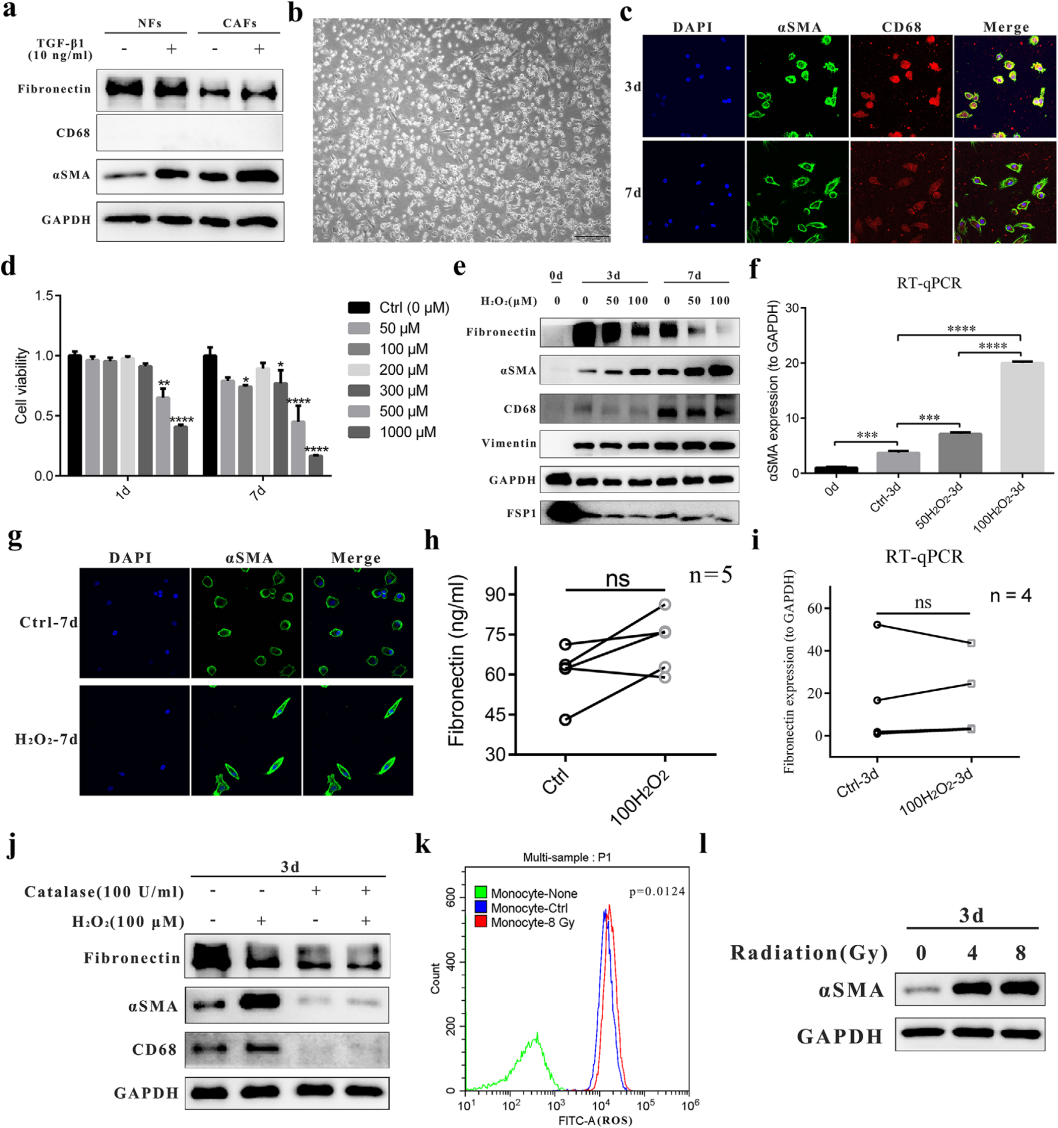
Oxidative stress induced MMT in vitro. A, Western blot analysis of fibronectin, CD68, and αSMA expression (3 days). GAPDH was used as the loading control. B, Representative photograph of human monocyte‐derived macrophages cultured in vitro (7 days). C, Representative immunofluorescence staining for αSMA (green), CD68 (red), and DAPI (blue, for nuclear staining) in monocytes cultured for 3 or 7 days in vitro. D, A CCK‐8 assay was used to evaluate the cytotoxicity of H_2_O_2_ to monocytes cultured in vitro for 1 or 7 days. Data from one representative donor of three donors are shown. Two‐way ANOVA followed by Bonferroni's post hoc test was used to evaluate the significance of the differences between the experimental and ctrl groups. E, Western blot analysis of αSMA, vimentin, FSP1, and CD68 expression. GAPDH was used as the loading control. F, Reverse transcription‐quantitative polymerase chain reaction (RT‐qPCR) measurement of αSMA gene expression levels. GAPDH was used as the loading control. One‐way ANOVA followed by Bonferroni's post hoc test was used to evaluate the significance of the differences between the groups. G, Immunofluorescence staining for αSMA (green), CD68 (red), and DAPI (blue, for nuclear staining) in macrophages treated or not with treated 100 µM H_2_O_2_. Cells treated with H_2_O_2_ tended to be spindle shaped. H, ELISA of fibronectin concentrations in supernatants from monocytes treated or not treated with 100 µM H_2_O_2_ (n = 5). A paired *t*‐test was used for comparisons. I, RT‐qPCR measurement of fibronectin gene expression levels (n = 4). The Wilcoxon matched‐pairs test was used for comparison. J, Western blot analysis of fibronectin, αSMA, and CD68 expression in monocytes treated with H_2_O_2_ (100 µM, ±) or the H_2_O_2_‐scavenging enzyme catalase (100 U/mL, ±) on day 3. GAPDH was used as the loading control. K, Flow cytometric analysis of intracellular ROS levels in monocytes irradiated with 8 Gy or sham‐irradiated, using H2DCFDA fluorescent probe (green, without probe; blue, sham‐irradiated; red, irradiated with 8 Gy). A paired *t*‐test was used for comparisons between Ctrl and 8 Gy groups (n = 3). L, Western blot analysis of αSMA expression on day 3 after irradiation (0/4/8 Gy). GAPDH was used as the loading control. The data are expressed as the means ± standard errors of the mean and are representative of at least three experiments Abbreviations: MMT, monocyte‐to‐myofibroblast transdifferentiation; NFs, normal fibroblasts; CAFs, cancer associated fibroblasts; DAPI, 4′,6‐diamidino‐2‐phenylindole; FSP1, fibroblast‐specific protein 1; RT‐qPCR, reverse transcription‐quantitative polymerase chain reaction; ELISA, enzyme‐linked immunosorbent assay; ns, not significant (*P* > .05). ^*^ *P* < .05; ^***^ *P* < .001; ^****^ *P* < .0001.

Because the precision of above analysis was limited owing to the close proximity of TAMs and CAFs in the stroma, we isolated fibroblasts in vitro^35^ and found that CAFs expressed higher levels of αSMA than NFs (Figure 2A). In addition, exogenous stimulation with TGF‐β1 induced αSMA expression by both NFs and CAFs. However, these cells did not express CD68, as assessed by WB or IF (Figures 2A, S1A, and S1B).

In addition, considering that macrophages have phagocytic capacity, to exclude the possibility of macrophages phagocytosing αSMA^+^ cells or debris, we isolated fresh human blood monocytes (>95% were CD14^+^; Figure S1C) from PBMCs. WB result showed that monocytes expressed almost no αSMA, fibronectin, or vimentin but did express FSP1. After differentiation in vitro (7 days, without other stimuli), most cells were fried egg shaped with a few being spindle shaped (Figure 2B), and cells started to express αSMA at a relatively low level (Figures 2C and 2E).

Then we explored the underlying mechanisms of αSMA expression. First, we speculated that cytokines secreted from PDAC cancer cells may play a role in MMT. However, compared with DMEM, supernatant from pancreatic cancer cells or HPDE6‐C7 had no obvious effect on αSMA expression by monocytes/macrophages (Figure S2A). Second, TGF‐β1 is the primary cytokine initiating fibrosis in many organs but did not obviously affect MMT (Figure S2B). Because ROS were generated during monocyte‐to‐macrophage differentiation, and macrophages expressed αSMA at a low level, we speculated that ROS were the primary cause of αSMA expression. Monocytes are vulnerable to oxidative stress,^41^ and H_2_O_2_ has been regularly adopted to assess the effects of oxidative stress. In this study, we performed a CCK‐8 assay to demonstrate short‐term (1 day) and long‐term (7 days) cytotoxicity (Figure 2D) and demonstrated that H_2_O_2_ concentrations generally not exceeding 100 µM were relatively nontoxic to monocytes/macrophages.

H_2_O_2_ significantly induced αSMA RNA and protein expression in a time‐ and concentration‐dependent manner (Figures 2E and 2F), suggesting that αSMA expression was regulated at the transcriptional level. Although αSMA expression in H_2_O_2_‐treated monocytes was lower than that in pancreatic fibroblasts, it was much higher than that in AsPC‐1, BXPC‐3, CFPAC‐1, PANC‐1, and HPDE6‐C7 (data not shown). Additionally, we demonstrated upregulated αSMA expression by single cell and morphological changes, with H_2_O_2_‐treated cells being spindle shaped (Figure 2G).

Other markers of myofibroblasts include fibronectin, Col1, and FSP1. In this study, we found that monocytes expressed high levels of FSP1; however, after differentiation in vitro, the expression of FSP1 in HMDMs decreased (Figure 2E). Regarding the secreted proteins fibronectin and Col1, freshly isolated monocytes expressed no fibronectin or Col1, and cells cultured in vitro started to express fibronectin (Figure S2C) but not Col1; however, the effect of H_2_O_2_ on fibronectin expression varied among cells from different donors, and the difference in mRNA (Figure 2I) or protein (Figures 2E, 2H, and 2J) levels did not reach statistical significance.

In addition, as shown in Figure 2J, after the addition of catalase, a key antioxidant enzyme responsible for the conversion of H_2_O_2_ to water and oxygen, monocytes/macrophages did not express αSMA, and catalase neutralized the effect of H_2_O_2_ on αSMA expression. In addition, CD68 was also not expressed in the catalase‐treated group, indicating that a low concentration of H_2_O_2_ (ie, ROS) was important for monocyte‐to‐macrophage differentiation. Intriguingly, catalase almost completely inhibited fibronectin production, as demonstrated by RT‐qPCR, ELISA (data not shown) and WB (Figure 2J), suggesting that basal H_2_O_2_ production was necessary for fibronectin generation.

Finally, to more accurately and precisely confirm the effect of oxidative stress, we adopted another method of oxidative stress induction, radiation, to verify the role of ROS in MMT.^42^ Radiation exhibited different levels of cytotoxicity to different donors (Figure S2D) and induced ROS production in monocytes (8 Gy; Figure 2K). As shown in Figure 2L, radiation increased αSMA expression, and this increase was also inhibited by catalase (data not shown).

### 3.3 MMT inhibited the phagocytic function of monocytes/macrophages

**FIGURE 3:**
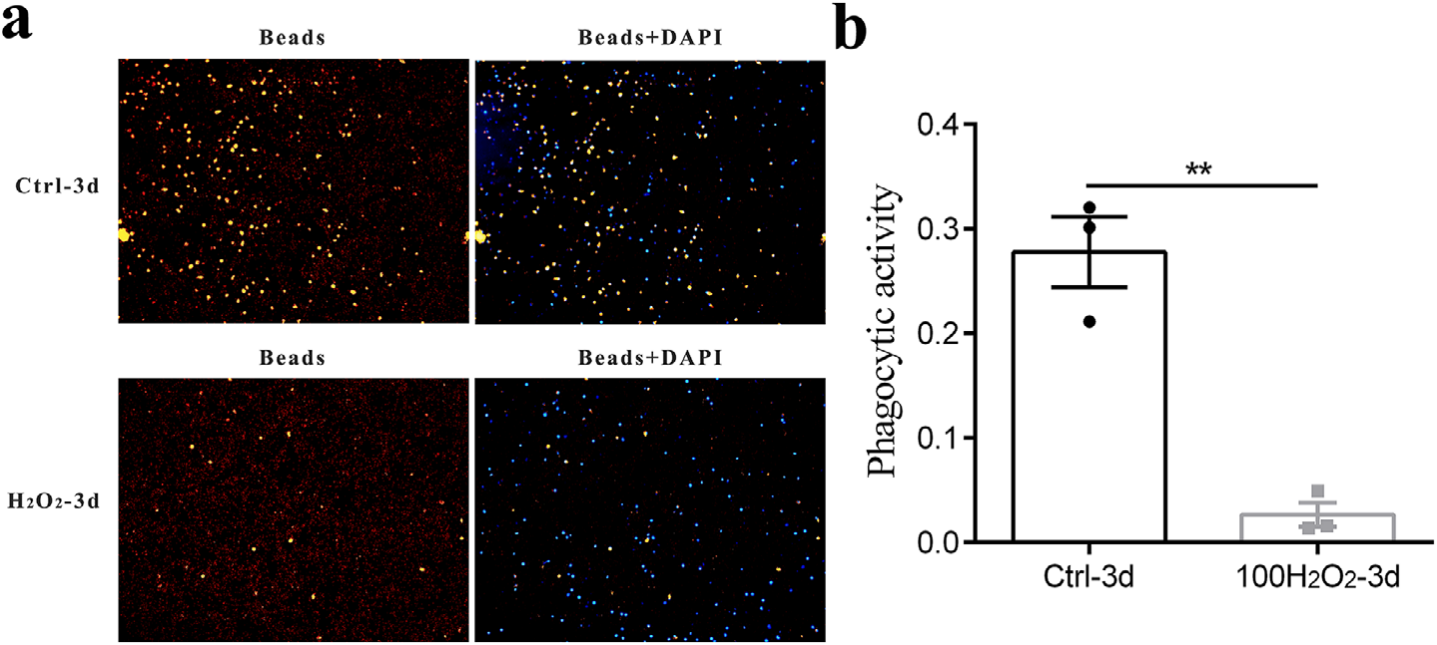
MMT inhibited the phagocytic function of monocytes/macrophages. A, Representative immunofluorescence images showing phagocytosis of latex beads (red) by monocytes treated or not treated with 100 µM H_2_O_2_ for 3 days. Nuclei were counterstained with DAPI (blue). B, Phagocytic capacity was quantified by measuring phagocytic activity (the number of beads divided by the number of nuclei) (n = 3). The data are expressed as the means ± standard errors of the mean. A paired *t*‐test was used for comparisons Abbreviations: MMT, monocyte‐to‐myofibroblast transdifferentiation; DAPI, 4′,6‐diamidino‐2‐phenylindole. ^**^ *P* < .01

Macrophages are innate immunocytes with phagocytic capacity; however, fibroblasts are generally thought to have no capacity for phagocytosis. Therefore, we evaluated the changes in phagocytic capacity during MMT. We used latex beads and found that treatment with 100 µM H_2_O_2_ significantly reduced the functional phagocytic capacity, as indicated by the phagocytic activity (Figure 3, *P* < .001).

### 3.4 The p53 stabilizer nutlin‐3 induced MMT through ROS generation but not through the p53 transcription/mitochondria‐dependent signaling pathway

**FIGURE 4:**
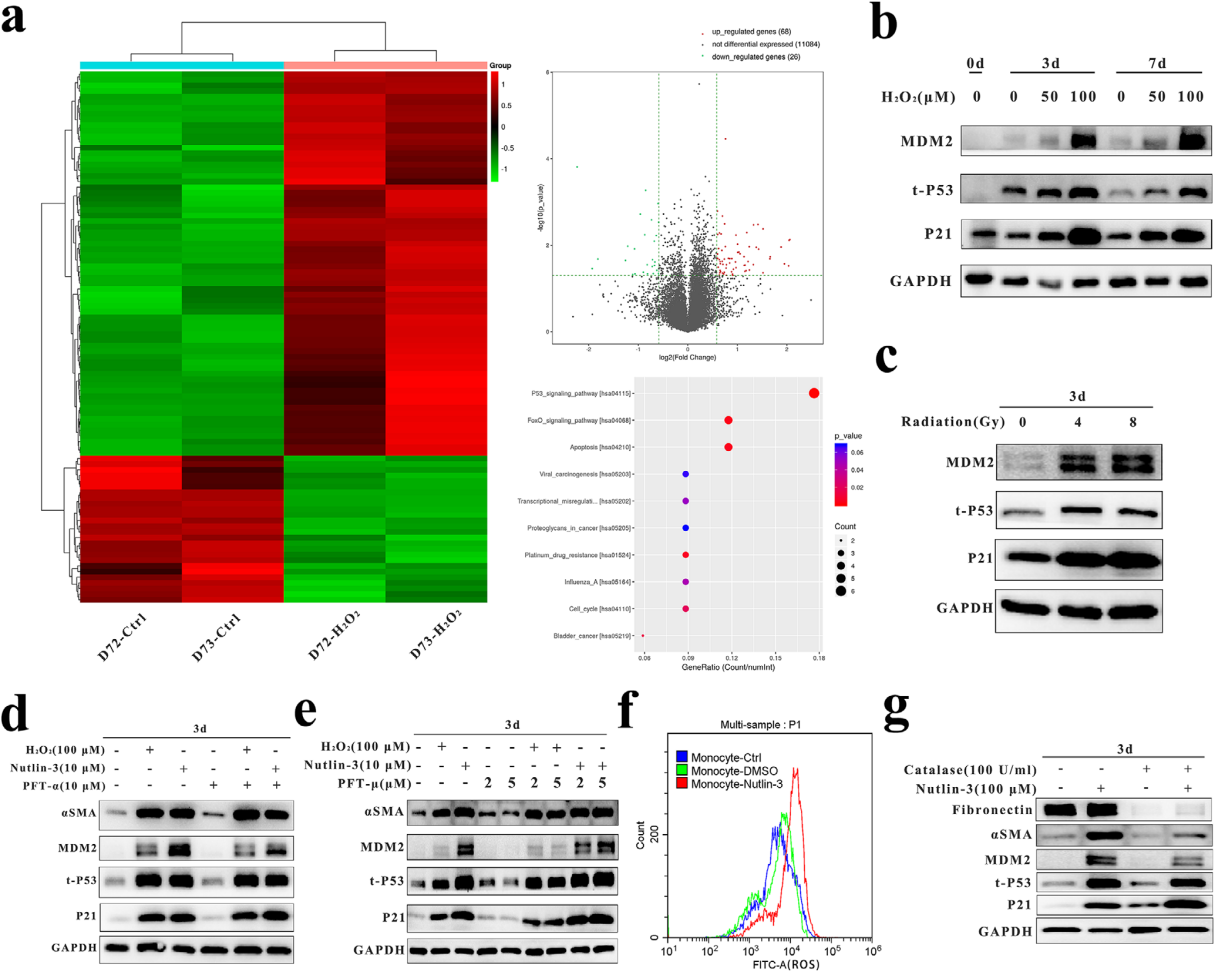
The p53 stabilizer nutlin‐3 induced MMT through ROS generation but not through the p53 transcription/mitochondria‐dependent signaling pathway. A, RNA‐seq analysis of monocytes obtained from two healthy donors (D72 and D73), and treated or not treated with 100 µM H_2_O_2_ for 7 days. The heatmap shows hierarchical clustering of mRNA levels of genes in monocytes treated as indicated; the volcano plot shows differentially expressed genes plotted as the log2(fold change) versus the –log10(*P*‐value). A total of 94 differentially expressed genes between the H_2_O_2_‐treated cell group and the control cell group exceeded the established thresholds (–log10(*P*‐value) > 1.30 and |log2‐fold change| > .585; upregulated genes are shown in red, and downregulated genes are shown in green). KEGG pathway analysis was performed on the upregulated genes identified by RNA‐seq. B and C, Western blot analysis of the expression of p53 and the target genes p21 and MDM2 in freshly isolated monocytes (0 day), monocytes treated with 0/50/100 µM H_2_O_2_ for 3/7 days, and monocytes treated with radiation (0/4/8 Gy) for 3 days. GAPDH was used as the loading control. D and E, Western blot analysis of the expression of αSMA, p53, p21, and MDM2 in monocytes treated or not treated with nutlin‐3, H_2_O_2_, PFT‐α, or PFT‐μ. GAPDH was used as the loading control. F, Representative flow cytometric analysis of intracellular ROS levels in monocytes treated or not treated with nutin‐3 (10 µM) or the same volume of DMSO (blue, ctrl; green, DMSO; red, nutlin‐3). G, Western blot analysis of monocytes treated or not treated with 10 µM nutlin‐3 or 100U/mL catalase. GAPDH was used as the loading control. Data are representative of three experiments Abbreviations: MMT, monocyte‐to‐myofibroblast transdifferentiation; RNA‐seq, RNA sequencing; DEGs, differentially expressed genes; KEGG, Kyoto Encyclopedia of Genes and Genomes; DMSO, dimethyl sulfoxide.

First, we performed RNA sequencing (RNA‐seq) to identify DEGs between the groups of cells treated or not treated with H_2_O_2_ (Table S2), including αSMA (Fold change, 2.98; *P* = .036), and signaling pathways involved in MMT. In Figure 4A, the heatmap illustrates DEGs between the two group of cells and the volcano plot shows 68 upregulated genes and 26 downregulated genes in H_2_O_2_‐treated monocytes. In addition, we used KEGG analysis to screen the pathways enriched in the DEGs and found that H_2_O_2_ regulates the p53 pathway.

Second, through WB analysis, we demonstrated that H_2_O_2_ increased the expression of p53 and the target genes p21 and MDM2 in a time‐ and concentration‐dependent manner (Figure 4B); the same trend was observed for radiation (Figure 4C). Consistent with the results of a previous study,^43^ the p53 stabilizer nutlin‐3 upregulated αSMA expression in monocytes (Figure 4D). Although reports have indicated that αSMA is a transcriptional target of p53^44^ and that p53 can increase ROS production through a mitochondrial pathway,^45^, ^46^ neither PFT‐α (10 µM) nor PFT‐μ (2 or 5 µM) reduced αSMA expression induced by H_2_O_2_ or nutlin‐3 (Figures 4D and 4E), even at concentrations as high as 50 µM. Besides that, we demonstrated that nutlin‐3 induced ROS production in human monocytes (Figure 4F), and catalase addition reduced the αSMA expression induced by nutlin‐3 but had little effect on p53, p21, or MDM2 expression (Figure 4G).

### 3.5 ROS induced MMT through the p38‐mitogen‐activated protein kinase (MAPK) signaling pathway

**FIGURE 5:**
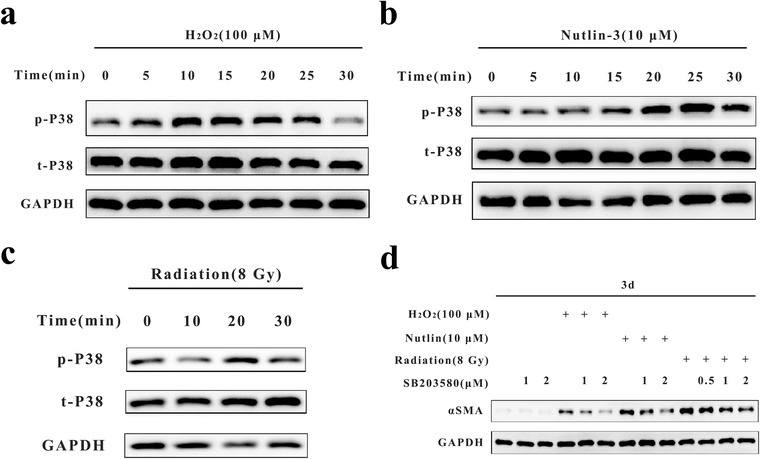
ROS induced MMT through p38‐MAPK signaling pathway. A‐C, Western blot analysis of p‐p38 and t‐p38 expression in monocytes treated with H_2_O_2_ (100 µM), nutlin‐3 (10 µM), or radiation (8 Gy) at different time points (≤30 min). GAPDH was used as the loading control. D, Western blot analysis of αSMA expression in monocytes treated with H_2_O_2_ (100 µM), nutlin‐3 (10 µM), or radiation (8 Gy) and the p38 inhibitor SB203580 (0/0.5/1/2 µM). GAPDH was used as the loading control. Data are representative of three experiments Abbreviations: MAPK, mitogen‐activated protein kinase.

Other studies showed that ROS can activate MAPKs in monocytes,^18^ and gene ontology analysis of RNA‐seq data identified MAPK kinase kinase activation by H_2_O_2_ (data not shown). We pretreated monocytes with different MAPK inhibitors and found that only the p38 inhibitor SB203580 had an inhibitory effect on ROS‐induced MMT. In this study, we found that 100 µM H_2_O_2_ activated p38 in 30 min, with the activation peak at 10‐15 min (Figure 5A). As for Nutlin‐3 (10 µM) also activated p38 in 30 min, but the activation peaked relatively later than for H_2_O_2_ (Figure 5B). Nutlin‐3 was dissolved in DMSO, and the same volume of DMSO had little effect on p38 activation (data not shown). In addition, radiation (8 Gy) activated p38 in 30 min (Figure 5C). However, the p38 inhibitor SB203580 (1‐2 µM) partially but not completely inhibited αSMA expression induced by H_2_O_2_, nutlin‐3, or radiation (Figure 5D), suggesting that other pathways may also participate in the induction of αSMA expression during MMT.

### 3.6 MMT generated a unique subset of myofibroblasts in PDAC and promoted the proliferation of PDAC cells

To date, studies have shown that CAFs in the tumor milieu are heterogeneous, and increasing attention has been devoted to identifying new markers expressed by special subtypes of CAFs. In this study, we aimed to more precisely define the characteristics of these transdifferentiated cells.

**FIGURE 6:**
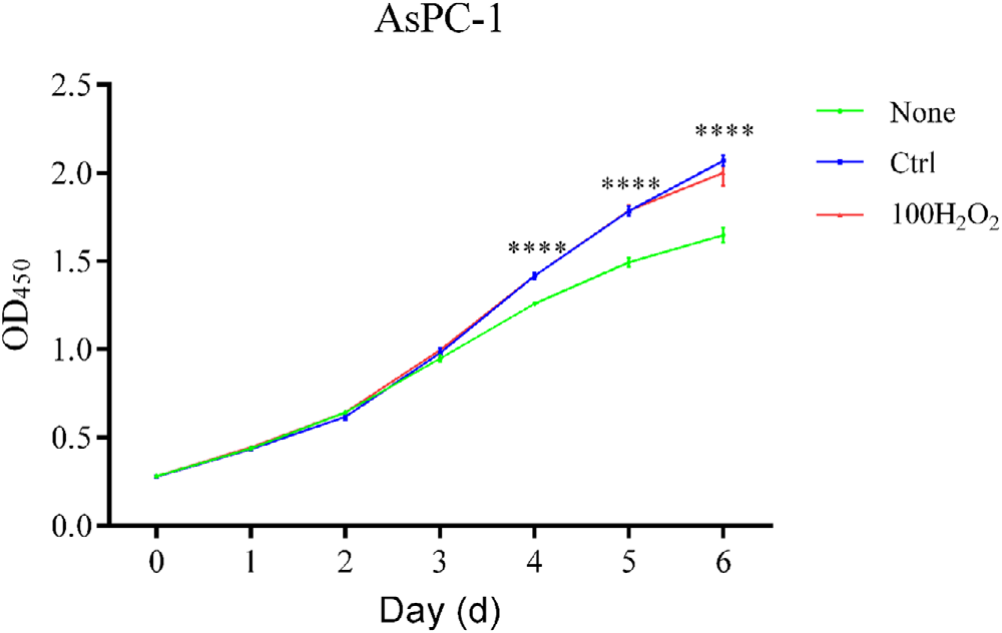
MMT generated a subset of myofibroblasts promoting the proliferation of cancer cells in PDAC. A CCK‐8 assay was used to evaluate the proliferation of cancer cells (AsPC‐1) cultured with DMEM or supernatant (30%) from monocytes treated or not treated with 100 µM H_2_O_2_. Data are expressed as the means ± standard errors of the mean (n = 3). Two‐way ANOVA followed by Bonferroni's post hoc test was used to evaluate the significance of differences among the groups. The asterisks indicate a significant difference between the “100H_2_O_2_” and “None” groups Abbreviations: MMT, monocyte‐to‐myofibroblast transdifferentiation; PDAC, pancreatic ductal adenocarcinoma. ^****^ *P* < .0001

Other studies identified a new subset of CAFs with characteristics of inflammatory cells, termed inflammatory cancer‐associated fibroblasts (iCAFs), in PDAC.^47^ Thus, we hypothesized that MMT may produce CAFs similar to those in this newly identified subset. Supernatant from these MMT cells promoted the proliferation of AsPC‐1 cells (Figure 6) but had little effect on BxPC‐3, CFPAC‐1, or PANC‐1 cells (data not shown).

## 4 DISCUSSION

The accurate definition of fibroblasts is incompletely elucidated. Fibroblasts are large spindle‐shaped cells but no single fibroblast‐specific immunocytochemical marker has been identified; therefore, several markers, including αSMA, FSP1, FAP, Col1, and fibronectin, are combined to distinguish them. Similarly, the CAFs in PDAC are heterogeneous with different markers.^10^, ^48^ The same characteristic applies to the numerous origins of CAFs, and accumulating evidence indicates that normal resident fibroblasts, fibrocytes, MSCs, adipocytes, and cancer cells can transdifferentiate into fibroblasts in different types of cancer.^11^, ^12^

A previous report demonstrated that mouse aortic smooth muscle cells transdifferentiated into a macrophage‐like state after cholesterol loading; this transdifferentiation was accompanied by decreased protein levels of αSMA and increased levels of CD68 and Mac‐2.^24^ Many studies have reported that TGF‐β1 can induce macrophage‐to‐myofibroblast transition in a kidney injury mouse model^32^, ^49^ and that monocytes can transdifferentiate into vascular endothelial cells^50^ or neuronal‐like cells.^51^ Here, we found for the first time that oxidative stress can induce MMT.

First, through IF we found costaining of αSMA with CD68 in PDAC tissues (Figure 1A), but αSMA expression in these cells was relatively lower than that in other CAFs. This expression pattern explained why only a few cells co‐expressed αSMA and CD68 in PDAC tissue as assessed by IF, considering that monocytes/macrophages cultured in vitro broadly expressed αSMA (Figures 2C and 2E). Consistent with another study showing that FSP1 could identify an inflammatory subpopulation of macrophages in the liver,^29^ we found that FSP1 colocalized broadly with CD68 in PDAC tissues (Figure 1B). In addition, through WB analysis, we found that freshly isolated human monocytes expressed high levels of FSP1 but that FSP1 expression declined during monocyte‐to‐macrophage differentiation (Figure 2E).

Ludin et al previously reported that monocytes/macrophages express αSMA, which preserves primitive hematopoietic cells in the bone marrow.^52^ Other studies demonstrated that TGF‐β1‐SMAD3 can induce MMT (αSMA and Col1 expression) in fibrotic diseases such as renal fibrosis.^32^ However, we found that TGF‐β1 activated fibroblasts (Figure 2A) but did not induce MMT in vitro (Figure S2B), as demonstrated also by another study.^43^ In addition, we found that αSMA expression by monocytes was not increased by exposure to tumor supernatant (Figure S2A). Intriguingly, although another study identified fibronectin expression in macrophages,^53^ we demonstrated here that monocytes began to express fibronectin but not Col1 upon differentiation to macrophages in vitro (Figures 2E and S2C), similar to the results of other studies demonstrating that tissue‐resident macrophages of embryonic origin produced much more Col1 than HMDMs in PDAC,^54^ and these CD68^+^Col1^−^ cells excluded the potential contamination with bone marrow‐derived fibrocytes.

Because ROS generation was low and αSMA was slightly upregulated during monocyte‐to‐macrophage differentiation, we speculated and demonstrated that ROS can increase the expression of αSMA by monocytes in vitro (Figure 2E). H_2_O_2_ is generally adopted to assess the effects of oxidative stress, and previous studies have detected the cytotoxicity of H_2_O_2_ to monocytes; however, the most commonly used interval was limited to 24 h.^18^ Therefore, we performed a CCK‐8 assay to demonstrate both short‐term (1 day) and long‐term (7 days) cytotoxicity and found that H_2_O_2_ concentrations generally not exceeding 100 µM were relatively nontoxic to monocytes/macrophages (Figure 2D). Furthermore, radiation was used to verify the role of ROS in MMT (Figure 2L), which could partially explain the radiation‐induced fibrosis in PDAC observed clinically.^55^ The contradiction between our results and those of previous studies^49^ may be explained by the fact that others explored the mechanisms mainly in mouse model of renal fibrosis, whereas we mainly used freshly isolated human monocytes cultured in vitro.

Additionally, previous studies have shown that moderate ROS levels are essential for monocyte‐to‐macrophage differentiation and phagocytic capacity of macrophages.^16^ In this study, we found a lack of expression of the macrophage differentiation marker CD68 after the addition of catalase (Figure 2J) and a significant reduction in phagocytic capacity during MMT induced by H_2_O_2_ (Figure 3), indicating an unfavorable effect of excessive ROS on the phagocytic function of macrophages. These results were consistent with those of a recently published study showing that macrophages can utilize excess ROS to stiffen the cytoplasm and reduce their phagocytic propensity,^56^ identifying ROS as a double‐edged sword in monocytes/macrophages.

Besides that, we identified DEGs through RNA‐seq and found that ROS can activate p53 (Figure 4A‐C). A previous study showed that nutlin‐3 can induce morphological changes and αSMA expression in human primary monocytes.^43^ PFT‐α has been reported to be a selective inhibitor of p53‐mediated transcription in the nucleus; PFT‐μ inhibits p53 binding to mitochondrial proteins. However, in our study, we demonstrated the role of nutlin‐3 in inducing αSMA expression, but neither PFT‐α nor PFT‐μ inhibited this MMT (Figures 4D and 4E), suggesting that nutlin‐3 induces MMT through a pathway other than the p53 transcription/mitochondria‐dependent signaling pathway. Another study reported that nutlin‐3 can induce ROS generation independent of the p53 status.^57^ In this study, we also demonstrated that nutlin‐3 promoted ROS generation in monocytes and that catalase inhibited nutlin‐3‐induced MMT (Figures 4F and 4G). However, we did not knock down p53 due to the low transfection efficiency of primary human monocytes, as also reported by others^58^; therefore, the precise mechanisms of nutlin‐3 or p53 in MMT merit further study.

In consistence with the results of other studies,^18^ we found that ROS can activate p38 in monocytes. Moreover, the p38 inhibitor SB203580 partially but not completely inhibited MMT (Figure 5), demonstrating that p38 plays a major role in the MMT process. However, other mechanisms may exist that need to be investigated.

In PDAC, ECM is a product of activated PSCs; however, the activity of PSCs as evaluated by αSMA expression is not always associated with the status of fibrosis,^59^ because αSMA expression is a more favorable marker for PSCs differentiation than for PSCs activity. The activated stroma index, that is, the ratio of the αSMA‐stained area to the Col1‐stained area, is a novel index and has been used to classify PDAC into four types with significantly different overall survival: dormant (low αSMA/high Col1), inert (low αSMA/low Col1), fibrogenic (high αSMA/high Col1), and fibrolytic (high αSMA/low Col1) PDAC.^60^, ^61^ Our results demonstrated MMT with elevated expression of αSMA and fibronectin but not Col1, suggesting that MMT contributes to the fibrolytic type, which has the worst prognosis among these types.

CAFs in PDAC are heterogeneous. David et al identified two distinct CAFs subtypes in PDAC: iCAFs, which lack elevated αSMA expression and instead express inflammatory markers such as IL‐6 and leukemia inhibitory factor and are located farther away from tumor cells; and myofibroblastic CAFs, which express markers of myofibroblasts, such as αSMA, and are found adjacent to tumor cells.^47^, ^48^ Recently, this group found another subtype of CAFs, antigen‐presenting CAFs, which express major histocompatibility complex class II and CD74 molecules but not classical costimulatory molecules.^62^ However, these studies have precluded CD45^+^ immune cells at isolating CAFs for single‐cell RNA‐seq. In our study, through RNA‐seq, we defined MMT cells as another type of CAFs, with characteristics of macrophages, in PDAC (Table S2). For example, we recently identified CD10 expression by monocytes or macrophages in PDAC,^34^ and other researchers have reported that CD10^+^ CAFs can promote the progression of PDAC and breast cancer.^63^, ^64^ In summary, MMT promotes the production of CD10^+^αSMA^+^ cells in cancers.

Finally, we explored the effect of MMT on cancer cells and found that supernatant from MMT cells could promote the proliferation of AsPC‐1 cells (Figure 6) but had little effect on other PDAC cancer cell lines, which may be attributed to the limited amount of freshly isolated monocytes and thus the low levels of cytokines in the supernatant. However, we did not conduct animal experiment, compare gene expression between monocyte‐derived myofibroblasts and PSC‐derived myofibroblasts, or explore the prognostic value of MMT, which needs further investigation in the future.

**FIGURE 7:**
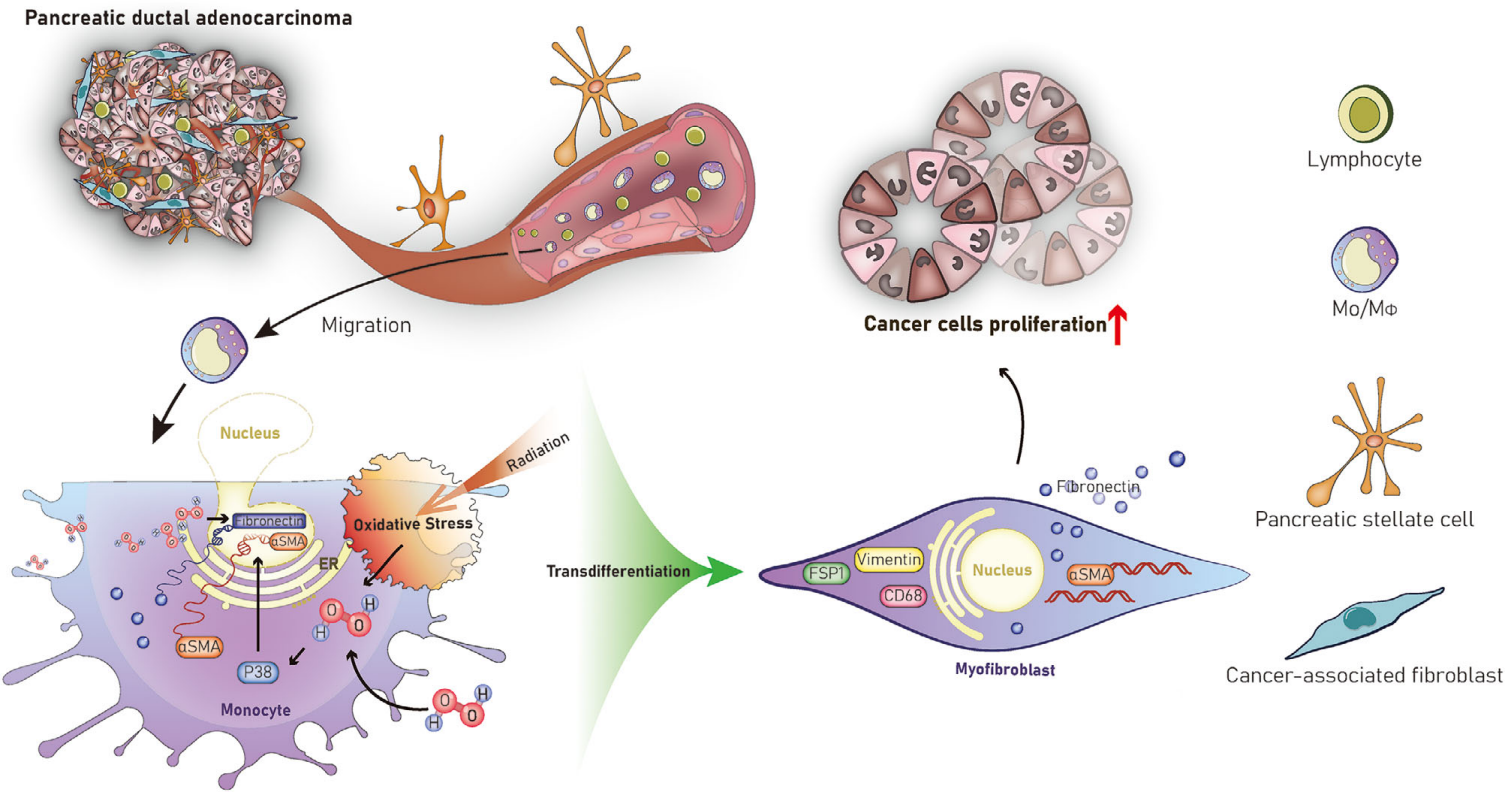
Schematic diagram of ROS‐dependent MMT in PDAC. Monocytes in human blood are recruited into PDAC tissues. Low intracellular levels of H_2_O_2_ are important for fibronectin mRNA synthesis, protein expression, and secretion in monocytes and macrophages. Oxidative stressors, such as radiation, can reduce the phagocytic capacity and simultaneously activate the p38‐MAPK signaling pathway, thus inducing αSMA mRNA and protein expression in monocytes/macrophages. However, these cells do not express Col1. Therefore, myofibroblasts transdifferentiated from monocytes are characterized by low phagocytic capacity; high αSMA/Col1 levels; fibronectin, vimentin, FSP1, and CD68 expression; and features of inflammatory cells. In addition, supernatant from these cells can stimulate cancer cell (AsPC‐1) proliferation, thus promoting tumor progression in PDAC. Abbreviations: MMT, monocyte‐to‐myofibroblast transdifferentiation; PDAC, pancreatic ductal adenocarcinoma; MAPK, mitogen‐activated protein kinase; Col1, collagen 1.

In conclusion, oxidative stress in the extracellular tumor microenvironment or inside cells could induce MMT in PDAC, thus inducing reactive stroma and modulating immunosuppression (Figure 7). Reducing oxidative stress may be a promising future therapeutic regimen, because other studies have found that antioxidant therapy blocks TAMs differentiation and tumorigenesis in mouse models of cancer.^16^

## CONFLICT OF INTEREST

The authors declare no conflict of interest.

## Supporting information


**Fig. S1**. Characteristics of primary isolated fibroblasts from patients with PDAC and primary isolated monocytes from healthy donors.
(a‐b) Immunofluorescence staining for αSMA (green) and DAPI (blue, for nuclear staining) in normal fibroblasts or cancer‐associated fibroblasts. A nonspecific IgG antibody was used as the negative control. (c) Flow cytometric analysis of CD14 expression by cells isolated from PBMCs, gated on viable cells by forward scatter and side scatter. An isotype control antibody was used as the negative control (red, staining with the anti‐CD14 antibody; blue, staining with the isotype control antibody). Ninety‐eight percent of the cells were CD14^+^. Data are representative of at least three experiments. DAPI, 4′,6‐diamidino‐2‐phenylindole; PBMCs, peripheral blood mononuclear cells.

**Fig. S2**. Effect of exogenous stimuli on monocytes.
(a‐b) Western blot analysis of αSMA expression in monocytes treated or not treated with supernatants from the pancreatic cell lines Aspc‐1, BxPC‐3, CFPAC‐1, PANC‐1 and HPDE6‐C7 (30%) or with TGF‐β1 (0‐5 ng/ml) for 7 d. GAPDH was used as the loading control. (c) Western blot analysis of cellular fibronectin expression in freshly isolated monocytes (0 d) and monocytes treated with 100 µM H_2_O_2_ at different time points (≤ 30 min). GAPDH was used as the loading control. (d) A CCK‐8 assay was used to evaluate the cytotoxicity of radiation (0, 2, 4, 8 or 16 Gy) to monocytes cultured *in vitro* for 3 d (n = 3). One‐way ANOVA followed by Bonferroni's post hoc test was used to evaluate the significance of the differences between the experimental and ctrl groups. Data are representative of at least three experiments. *, P < 0.05.

**Table S1**. Primer sequences used for qPCR analysis.

**Table S2**. Differentially expressed genes between macrophages treated with or without 100 µM H_2_O_2_.
