# Supplementary figures and images for: Oxidative stress induces monocyte‐to‐myofibroblast transdifferentiation through p38 in pancreatic ductal adenocarcinoma
Source: Clin Transl Med. 2020 Jun 4;10(2):e41. doi: 10.1002/ctm2.41 (PMC7403727; doi:10.1002/ctm2.41)

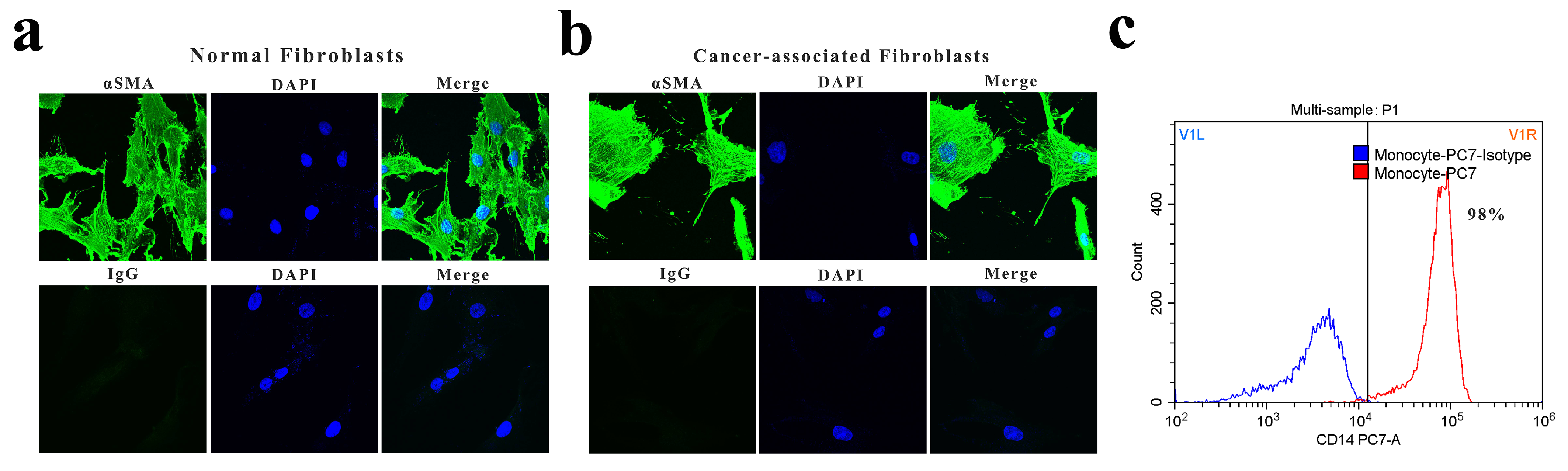

Supplement: Supplementary file 1 — Fig. S1. Characteristics of primary isolated fibroblasts from patients with PDAC and primary isolated monocytes from healthy donors. (a‐b) Immunofluorescence staining for αSMA (green) and DAPI (blue, for nuclear staining) in normal fibroblasts or cancer‐associated fibroblasts. A nonspecific IgG antibody was used as the negative control. (c) Flow cytometric analysis of CD14 expression by cells isolated from PBMCs, gated on viable cells by forward scatter and side scatter. An isotype control antibody was used as the negative control (red, staining with the anti‐CD14 antibody; blue, staining with the isotype control antibody). Ninety‐eight percent of the cells were CD14+. Data are representative of at least three experiments. DAPI, 4′,6‐diamidino‐2‐phenylindole; PBMCs, peripheral blood mononuclear cells. [file CTM2-10-e41-s001.tif]

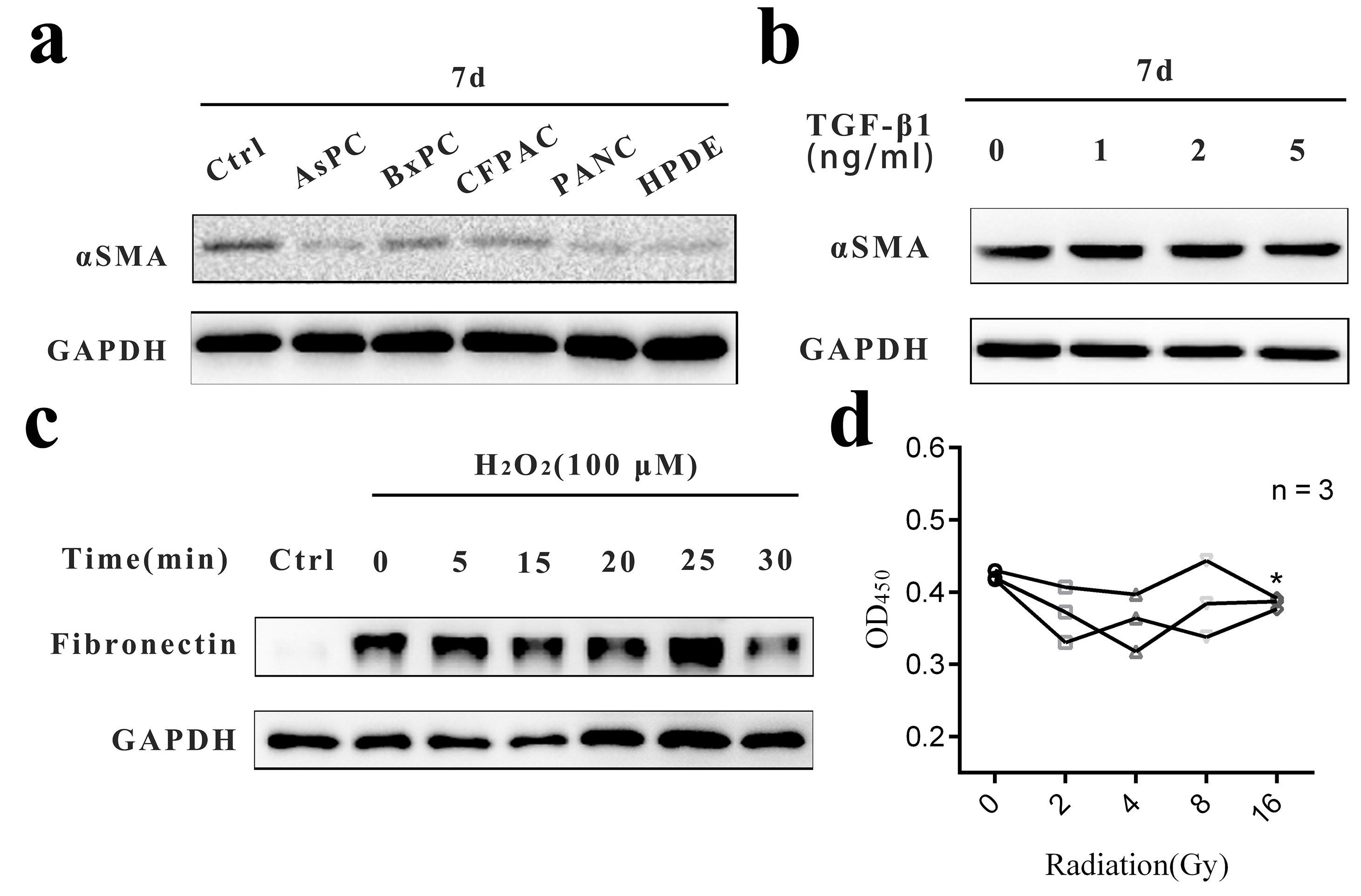

Supplement: Supplementary file 2 — Fig. S2. Effect of exogenous stimuli on monocytes. (a‐b) Western blot analysis of αSMA expression in monocytes treated or not treated with supernatants from the pancreatic cell lines Aspc‐1, BxPC‐3, CFPAC‐1, PANC‐1 and HPDE6‐C7 (30%) or with TGF‐β1 (0‐5 ng/ml) for 7 d. GAPDH was used as the loading control. (c) Western blot analysis of cellular fibronectin expression in freshly isolated monocytes (0 d) and monocytes treated with 100 µM H2O2 at different time points (≤ 30 min). GAPDH was used as the loading control. (d) A CCK‐8 assay was used to evaluate the cytotoxicity of radiation (0, 2, 4, 8 or 16 Gy) to monocytes cultured in vitro for 3 d (n = 3). One‐way ANOVA followed by Bonferroni's post hoc test was used to evaluate the significance of the differences between the experimental and ctrl groups. Data are representative of at least three experiments. *, P < 0.05. [file CTM2-10-e41-s002.tif]
