# Supplementary material for: Oxidative stress induces monocyte‐to‐myofibroblast transdifferentiation through p38 in pancreatic ductal adenocarcinoma
Source: Clin Transl Med. 2020 Jun 4;10(2):e41. doi: 10.1002/ctm2.41 (PMC7403727; doi:10.1002/ctm2.41)
Supplement: Supplementary file 3 — Table S1. Primer sequences used for qPCR analysis. [file CTM2-10-e41-s003.docx]

**Table S1.** Primer sequences used for qPCR analysis.

|  | Forward primer | Reverse primer |
| --- | --- | --- |
| αSMA | TGGCTATTCCTTCGTTACTACTGCT | CATCAGGCAACTCGTAACTCTTCTC |
| Fibronectin | ACAACACCGAGGTGACTGAGAC | GGACACAACGATGCTTCCTGAG |
| Col1a1 | GATTCCCTGGACCTAAAGGTGC | AGCCTCTCCATCTTTGCCAGCA |
| GAPDH | GTCTCCTCTGACTTCAACAGCG | ACCACCCTGTTGCTGTAGCCAA |
